# A Systematic Review of Post-Work Core Temperature Cooling Rates Conferred by Passive Rest

**DOI:** 10.3390/biology12050695

**Published:** 2023-05-09

**Authors:** Matt Brearley, Rachel Berry, Andrew P. Hunt, Rodney Pope

**Affiliations:** 1 Thermal Hyperformance, Hervey Bay, QLD 4655, Australia; 2 National Critical Care and Trauma Response Centre, Darwin, NT 0800, Australia; 3 School of Allied Health, Exercise & Sports Sciences, Charles Sturt University, Albury, NSW 2640, Australia; 4 School of Biomedical Sciences, University of New South Wales, Sydney, NSW 2052, Australia; 5 School of Biomedical Sciences, Faculty of Health, Queensland University of Technology (QUT), Brisbane, QLD 4059, Australia; 6 Tactical Research Unit, Bond University, Robina, QLD 4229, Australia

**Keywords:** cooling, heat stress, hyperthermia, passive rest, recovery, WBGT, work

## Abstract

**Simple Summary:**

Despite recommendations for, and the prevalence of, passive rest amongst heat-exposed occupational groups to mitigate heat stress, there is limited information regarding the effectiveness of this control measure. This systematic review of post-work core temperature cooling rates conferred by passive rest reports that 8 of the 50 included datasets failed to indicate cooling. Of the remaining 42 datasets, only 10 indicated core temperature cooling rates exceeding 0.034 °C min^−1^ or ~0.5 °C per 15 min, with participants wearing athletic attire or similar in each of these studies. Cooling during passive rest while wearing more insulative work attire or similar was only achieved in 7 of 13 datasets. Irrespective of its widespread implementation, these findings indicate that passive rest does not reverse the elevated core temperatures of heat-exposed workers in a timely manner. Alternative cooling methods are required to mitigate heat stress now and into the future.

**Abstract:**

Physical work increases energy expenditure, requiring a considerable elevation of metabolic rate, which causes body heat production that can cause heat stress, heat strain, and hyperthermia in the absence of adequate cooling. Given that passive rest is often used for cooling, a systematic search of literature databases was conducted to identify studies that reported post-work core temperature cooling rates conferred by passive rest, across a range of environmental conditions. Data regarding cooling rates and environmental conditions were extracted, and the validity of key measures was assessed for each study. Forty-four eligible studies were included, providing 50 datasets. Eight datasets indicated a stable or rising core temperature in participants (range 0.000 to +0.028 °C min^−1^), and forty-two datasets reported reducing core temperature (−0.002 to −0.070 °C min^−1^) during passive rest, across a range of Wet-Bulb Globe Temperatures (WBGT). For 13 datasets where occupational or similarly insulative clothing was worn, passive rest resulted in a mean core temperature decrease of −0.004 °C min^−1^ (−0.032 to +0.013 °C min^−1^). These findings indicate passive rest does not reverse the elevated core temperatures of heat-exposed workers in a timely manner. Climate projections of higher WBGT are anticipated to further marginalise the passive rest cooling rates of heat-exposed workers, particularly when undertaken in occupational attire.

## 1. Introduction

Energy expenditure can increase up to 25 times from resting levels during strenuous physical activity, requiring a considerable elevation in metabolic rate and in turn, causing body heat production. The elevation of metabolic heat production during periods of labour-intensive work produces a concomitant activation of heat loss mechanisms in an effort to achieve thermal balance, stabilise body temperature, and minimise heat stress [1], with the latter defined as the net heat load to which an individual is exposed [2]. When heat dissipation fails to match body heat production, body heat is stored, and this is reflected by an increase in core temperature (T_c_). If body heat storage is sustained such that T_c_ is elevated for prolonged periods or reaches hyperthermic levels, it may overwhelm an individual’s thermal tolerance, with implications for physical [3] and mental performance [4], potentially contributing to accidents and injuries [5] and to exertional heat-related illnesses, which can ultimately be fatal [6].

Balancing physical workload and heat loss to prevent hyperthermia and associated sequalae is a routine challenge for heat-exposed workers, including those employed within industry, emergency response, law enforcement, and the military. Situations that compromise self-pacing and the mandatory protective attire worn by these workers further complicate their thermal challenge. Clothing acts as a barrier to body heat exchange with the environment [7]; however, the maximal potential for body heat dissipation is a product of the prevailing environmental conditions [8]. This is an important consideration, as both clothing ensembles that provide greater protection and harsher environmental conditions exacerbate heat stress.

To assist workers in managing their heat stress on a day-to-day basis, most organisations implement a range of heat stress controls, with heat stress indices and passive rest periods amongst the most practical and popular options [9]. The most frequently utilised index of heat stress is the Wet-Bulb Globe Temperature (WBGT) [10], which was developed by the US military in the 1950s to limit heat-related illness during recruit training [11]. It continues to be utilised by many militaries and is also embedded within the guidance from peak workplace organisations, including the American Conference of Governmental Industrial Hygienists (ACGIH) [12]. The WBGT combines measures of ambient temperature, atmospheric moisture, and solar radiation to classify the environmental conditions according to the expected levels of heat stress during exposure and, when combined with the classification of physical workload being undertaken by personnel, WBGT categories recommend durations of work and rest periods [12].

Through the cessation of physical activity (otherwise known as passive rest), removing protective clothing, and seeking shade or a cooler environment, passive rest periods reduce metabolic heat production and may provide an opportunity for personnel to dissipate body heat. Recognition of rest periods as a fundamental heat stress control is evidenced by their incorporation into recommendations from heat stress indices [13]. While rest periods are an embedded practice in many workplaces, their effectiveness for lowering T_c_ is determined by factors that impact body heat dissipation, such as work uniforms, and the capacity of the environment for heat and moisture exchange. In regard to the latter, ACGIH recommendations for rest as a proportion of work periods increase in a step-wise manner as WBGT increases [12]. Furthermore, projected WBGT classifications for coming decades suggest workers will encounter greater heat exposure [14].

In response to the evolving risk profile of heat-exposed workers, active cooling controls to expedite the reduction of T_c_ have been developed [15,16]. While some of these methods demonstrate superiority compared to passive rest, their application in many workplaces may not be deemed necessary, nor feasible, particularly for those in resource-limited settings. On this basis, it is foreseeable that passive rest will remain a key heat stress control into the future, even though climate projections indicate conditions will be less conducive to heat loss via passive rest. Yet, T_c_ cooling rates during passive rest alone have not been systematically compiled and reported, potentially limiting evidence-based management of worker heat stress. Therefore, this systematic review reports the post-work T_c_ cooling rates (°C min^−1^) conferred by passive rest to determine whether this strategy reverses elevated T_c_ in a timely manner across a range of environmental conditions.

## 2. Material and Methods

A systematic review was conducted to address the research aim. The review was registered with PROSPERO prior to the completion of preliminary searches and commencement of data extraction (PROSPERO 2021 CRD42021259757).

### 2.1. Search Strategy

PubMed, Scopus, Cumulative Index to Nursing and Allied Health Literature (CINAHL), SPORTDiscus, and Web of Science databases were searched from database inception to 31 May 2021, for articles containing the following MeSH (or equivalent controlled vocabulary) terms and keywords: (“cooling rate”, “cooling”, “water immersion”, “cold air”, “cold room”, “cool room”, “cool air” “ice vest”, “ice jacket”, “cool vest”, “cool jacket”, “crushed ice ingestion”, “ice vest”, “neck collar”, “neck cooling”, “ice slurry”, “ice slush”, “heat mitigation”, “air vest”, “post-exercise”, “postexercise”, “passive rest”, “passive cool”, “shade”, “control trial”, “resting”, “recovery”) AND (“hyperthermia”, “heat stroke”, “heat exhaustion”, “heat stress”) AND (“core temperature”, “core body temperature”, “rectal temperature”, “gastrointestinal temperature”). Results were restricted to English language. Review articles and reference lists were searched and cross-referenced to identify additional reports of primary research for possible inclusion. The listed search terms relating to various cooling interventions were included to enable identification of studies where T_c_ changes had been assessed and reported, since many such studies reported T_c_ changes that accompanied passive rest (as a control condition) in addition to reporting temperature changes resulting from a cooling intervention. Note that use of the SCOPUS database was not initially planned or included in our PROSPERO registration. However, to complement the other databases and to ensure our search was as comprehensive as possible, the SCOPUS database was subsequently included.

### 2.2. Inclusion and Exclusion Criteria

Primary inclusion criteria were: 1. controlled trials (randomised or non-randomised) involving non-clinical human participants aged 18–50 years; 2. participating in physical activity to induce an elevated T_c_ (minimum of 38.2 °C/100.8 °F); and 3. measurement of T_c_ at the rectum, gastrointestinal tract, or oesophagus. Secondary (or specific) inclusion criteria were: 4. that each participant undertook passive cooling (no physical activity) for a minimum period of 15 min in one or more conditions observed in the study; 5. that reported data permitted extraction or calculation of T_c_ changes that occurred during the passive cooling period; and 6. that reported data permitted extraction or calculation of WBGT (minimum of ambient temperature and relative humidity reported).

Review articles, abstracts, case studies, editorials, and literature not written in the English language were excluded. Studies in which either the environmental conditions assessment or T_c_ measurement system were scored as having low quality (see Quality Assessment section below) were also excluded.

### 2.3. Screening and Selection

Following the literature search, titles and abstracts of identified articles were screened by the lead author, with reference to the study eligibility criteria, and clearly ineligible articles were removed. Following this screening process, full-text copies of remaining articles were retrieved. These full-text articles were then reviewed in detail by each of two reviewers (MB, RB), independently, to assess their eligibility for inclusion. Decisions of each reviewer regarding eligibility of the full-text articles were discussed by the two reviewers, and differences were resolved by consensus. The search, screening, and selection processes were performed according to the Preferred Reporting Items for Systematic Reviews and Meta-Analyses (PRISMA) [17].

### 2.4. Quality Assessment

Since the key variables of interest in this review were those related to environmental conditions and T_c_, quality assessment of the 44 included studies focused on the validity of environmental conditions assessment (particulars of the environmental monitoring system) and T_c_ measurement, specifically particulars of the instrumentation (and T_c_ monitoring system) used in each study. For this purpose, a three-classification rating system to indicate the assessed quality of these measures (high, low, or unclear) was utilised. A rating of unclear was applied where insufficient information was provided regarding the particulars of the environmental monitoring system or T_c_ monitoring system, respectively. A rating of high was applied where the respective system was confirmed as being widely utilised within research and/or industry settings by a thermal physiologist (MB). Note that the inclusion criteria of T_c_ measurement at the rectum, gastrointestinal tract, or oesophagus excluded invalid measurement T_c_ measurements sites, most notably tympanic temperature [18], prior to quality assessment. While the quality assessment deviated from the original intention documented in the PROSPERO registration, it was deemed the most relevant approach, given the aim of the review and most likely sources of bias. Studies in which the quality of the environmental monitoring and/or T_c_ monitoring systems was deemed to be low were excluded from the review.

### 2.5. Data Extraction

The following information was extracted from each included study: study location, number of participants, participant characteristics (sex, age, height, body mass, body fat, VO_2_max, heat acclimatisation status), environmental conditions (ambient temperature, relative humidity, solar radiation, wind speed, WBGT), passive rest characteristics (clothing worn, rest position, location, time between physical activity and commencement of passive rest, duration of passive rest), T_c_ measurement site, T_c_ at end of physical activity, T_c_ at start and cessation of passive rest, and T_c_ cooling rate per minute.

For studies where T_c_ cooling rate was not reported or could not be directly calculated from data presented in the text or tables, Web Plot Digitiser (https://automeris.io/WebPlotDigitizer, accessed on 16 January 2023) was utilised to extract T_c_ data from graphs for T_c_ cooling rate calculation.

### 2.6. Data Analysis and Treatment

Where T_c_ cooling rate was not provided, it was calculated as the difference between the T_c_ at the commencement and cessation of passive rest divided by the duration (min) of passive rest. To mitigate the impact of extended cooling durations reducing overall cooling rate [19], calculations of T_c_ cooling rate of were based on a cooling duration of 60 min, or less if observations were for a shorter period. For datasets that exceeded this duration, T_c_ cooling rate was calculated at the 60-min time-point, where possible.

T_c_ cooling rate was classified according to: <0.070 °C min^−1^ unacceptable; 0.070–0.100 °C min^−1^ acceptable; and >0.100 °C min^−1^ ideal [20].

To account for the insulative properties of clothing worn during passive rest, where clothing insulation values were not reported, insulation values were calculated according to estimates from the American Society of Heating, Refrigerating, and Air-Conditioning Engineers [21]. Clothing insulation (clo) values were classified as either low (clo < 0.40) or high (clo ≥ 0.40) rather than being based upon the often-used categories of “athletic” or “work” attire, as some athletic uniforms, such as those worn by American football athletes, produce clothing insulation values similar to industrial, emergency responder, and military uniforms [7].

Where the WBGT (°C) of the passive rest environment was not reported, it was calculated from environmental conditions according to the methods of Bernard and Pourmoghani [22] through the use of an Excel spreadsheet (https://www.climatechip.org/excel-wbgt-calculator, accessed on 12 September 2022). Ambient temperature (°C) and relative humidity (%) were required as a minimum for these calculations, with wind speed (m/s) also utilised where provided. In these calculations, globe temperature was assumed to be equal to ambient temperature where not provided, as all passive rest periods included in this review were undertaken in shaded outdoor or indoor locations.

The WBGT levels reported from each dataset were classified according to the American Conference of Governmental Industrial Hygienists [12] and the US Department of the Army [23] (Table 1).

Where not directly provided, body mass index [24] and body surface area [25] were also calculated from reported data.

Following reporting of this initial descriptive analysis of data from the included studies, a comparison was conducted, using an independent samples *t*-test, of the means of the mean T_c_ rates of change reported across studies in which participants wore attire with either a *low* or *high* clothing insulation factor. Exploratory Spearman’s correlation analyses were also subsequently conducted to investigate associations across the included studies between mean rates of change in T_c_ and potential predictors of this cooling rate, the latter including WBGT and reported participant characteristics.

## 3. Results

### 3.1. Search Results

**Figure 1:**
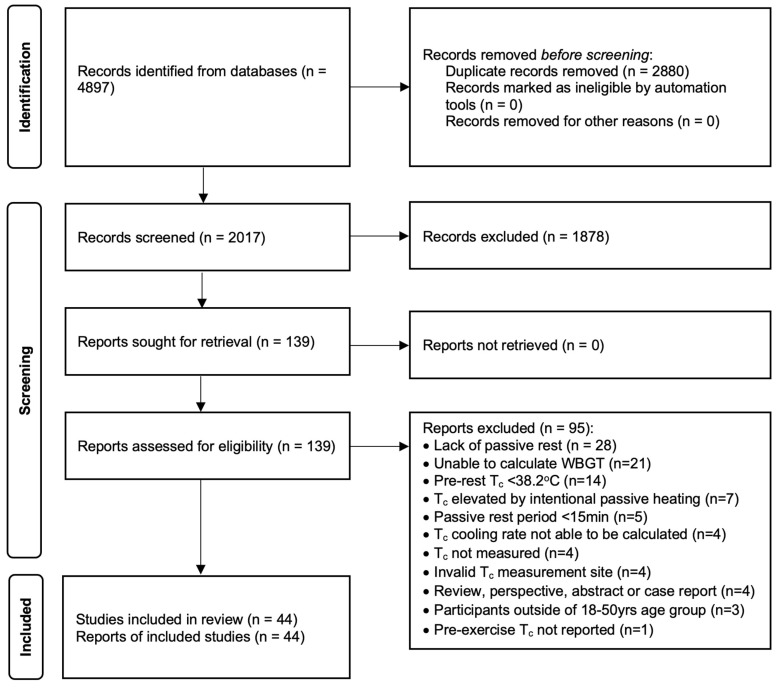
Preferred Reporting Items for Systematic Reviews and Meta-Analyses (PRISMA) flow diagram [17] detailing the results of the search, screening and selection process for the systematic review.

The initial literature search yielded 4897 references, and after removing duplicates and articles identified as clearly ineligible through screening of titles and abstracts, 139 full texts of the remaining articles were obtained. Following full-text review, 44 studies were deemed eligible for inclusion and were used in the analysis as shown in the Preferred Reporting Items for Systematic Reviews and Meta-Analyses (PRISMA) flowchart (Figure 1). Based on a full-text review, 95 studies were excluded, with 70 excluded based upon at least one of these four criteria: lack of passive cooling (28), unable to determine WBGT (21), T_c_ less than 38.2 °C upon passive rest commencement (14), and T_c_ elevated by intentional passive heating interventions (7).

### 3.2. Quality Assessment

The system for monitoring environmental conditions was infrequently reported, resulting in an unclear rating being applied to 36 of the included studies, comprising 42 datasets. The eight studies (eight datasets) that provided sufficient information to enable quality assessment were all deemed to use a valid system deemed to be of high quality. While the particulars of the instrumentation for T_c_ measurement were adequately detailed for all 44 studies, there were six studies (seven datasets) that provided insufficient information regarding the T_c_ monitoring system for quality assessment, resulting in a rating of unclear being assigned when assessing the validity of the T_c_ monitoring system for those studies. The remaining 38 studies (43 datasets) had validity of the instrumentation for T_c_ measurement rated as high.

### 3.3. Characteristics of Included Studies

A single dataset was extracted from 38 of the included studies [19,26,27,28,29,30,31,32,33,34,35,36,37,38,39,40,41,42,43,44,45,46,47,48,49,50,51,52,53,54,55,56,57,58,59,60,61,62], with the remaining six studies yielding two datasets each [63,64,65,66,67,68]. These 50 datasets from 44 included studies reported findings from a total of 562 participants (461 male, 84 female, 17 sex not reported) who undertook passive cooling. Characteristics of the included study datasets are summarised in Table 2. Passive rest was undertaken indoors (45 datasets) or in shaded outdoor conditions (five datasets), with participants generally in a seated position (36 datasets). Core temperature was measured at the rectum (30 datasets), gastrointestinal tract (16 datasets), or oesophagus (4 datasets). Time to commence passive rest following exercise was zero min (17 datasets), up to and including two min (9 datasets), from over two to six min (13 datasets), greater than six min to a maximum of 10 min (3 datasets), or not reported (eight datasets). Mean T_c_ upon commencement of the 15–75 min rest periods used across the included studies were in the range of 38.2–39.8 °C.

### 3.4. Core Temperature Cooling Rate

Eight datasets indicated stable or *rising* T_c_ in participants during passive rest (range 0.000 to 0.028 °C min^−1^), with the remaining 42 datasets reporting T_c_ cooling occurred at rates of −0.002 to −0.070 °C min^−1^ (Table 2). Table 3 indicates the number (*n*) of included datasets within each WBGT category, along with weighted mean rest durations, pre-rest T_c_, and change in T_c_ observed during the passive rest periods, across the datasets in each WBGT category. The 37 datasets arising from passive rest with attire considered to have *low* clothing insulation values are summarised in Table 4. Weighted mean changes in T_c_ were −0.012 to −0.041 °C min^−1^ across WBGT categories. The 13 datasets arising from passive rest with attire considered to have *high* clothing insulation values produced weighted mean T_c_ changes of 0.010 °C min^−1^ (indicating a mean heating effect) to −0.018 °C min^−1^ across WBGT categories (Table 5). On the basis of T_c_ cooling rate, one dataset was classified as reporting an acceptable T_c_ cooling rate with the T_c_ cooling rates reported in the remaining 49 datasets classified as unacceptable.

Consistent with these findings, exploratory analyses indicated that, across the included studies, the mean rates of cooling indicated by mean rates of change in T_c_ (°C min^−1^) were on average nearly eight times as high in the studies where participants wore attire with a low clothing insulation factor than in the studies where participants wore attire with a high clothing insulation factor (−0.027 °C min^−1^ vs. −0.004 °C min^−1^, respectively; t(48) = −4.91, *p* < 0.001). Across the studies involving participants wearing attire with *low* clothing insulation factors, no significant associations were evident between mean rates of change in T_c_ (°C min^−1^) and WBGT temperature (°C) or mean age, height, body mass, body fat level, BMI, body surface area, or pre-rest T_c_ of participants, where the studies reported these variables. In contrast, across the studies involving participants wearing attire with *high* clothing insulation factors, mean rates of change in T_c_ (°C min^−1^) in the 13 datasets that reported these variables were significantly (*p* ≤ 0.01) and positively associated with mean body mass (r*_s_*(11) = 0.75), BMI (r*_s_*(11) = 0.68), and BSA (r*_s_*(11) = 0.72) of the study participants, and these three predictor variables were also highly correlated with each other (r*_s_*(11) = 0.81 to 0.95, *p* < 0.01). These positive associations indicate that, as mean body mass or BMI or BSA increased, the mean T_c_ rate of cooling decreased or, where T_c_ rose during rest, the mean T_c_ rate of heating increased, since a positive change in T_c_ indicated a shift towards the heating end of the cooling-to-heating continuum. However, across these studies involving participants wearing attire with *high* clothing insulation factors, no significant associations were evident between mean rates of change in T_c_ (°C min^−1^) and WBGT temperature (°C) or mean age, height, body fat level, or pre-rest T_c_ of participants.

## 4. Discussion

This systematic review reports post-work T_c_ cooling rates conferred by passive rest across a range of environmental conditions. The primary finding was that post-work passive rest *while wearing occupational attire or similar* clothing produced mean rates of change in T_c_ of −0.018 to +0.010 °C min^−1^ (the latter indicating a passive *heating* rather than cooling effect) across all WBGT categories represented in the included studies (Table 5). This finding indicates that passive rest would *not* reverse the elevated T_c_ (≥38.2 °C) of heat-exposed workers [70,71,72] in a timely manner. In fact, it would take a minimum of 56 min of passive rest while dressed in occupational attire or similar to reduce T_c_ by 1.0 °C and in some circumstances, passive rest would not result in cooling at all but, instead, in further heating. Marginally better rates of cooling (ranging up to −0.070 °C min^−1^) were evident during passive rest in a few datasets where participants wore minimal clothing such as underwear or athletic attire (Table 4); however, such findings were inconsistent. At best, this would mean approximately 14 min of passive rest in minimal attire would be required to reduce T_c_ by 1.0 °C. However, in many circumstances, the required rest period to achieve the same reduction in T_c_ while dressed in minimal attire would be substantially longer.

While passive rest when encapsulated in protective clothing failed to lower T_c_ in several datasets [55,56,64], it is notable that in some cases, even when athletic attire [40], work pants only [68], or work pants and cotton t-shirt were worn [55,56], T_c_ was observed to *rise* during passive rest, despite incomplete coverage of the body surface area by clothing (Table 2). Commencing passive rest immediately following cessation of intense physical activity may contribute to rising T_c_ during the rest period, as metabolic rate and therefore body heat production remains elevated. Although some datasets reported a delay between physical activity cessation and commencement of passive rest due to participant relocation, instrumentation or similar, 17 datasets stated that commencement of passive rest occurred immediately upon cessation of physical activity [40,64,68]. However, surprisingly there was no indication in the data from the included studies that T_c_ cooling rates were consistently poorer at higher WBGT classifications, and the lack of such a finding—which might intuitively be expected—does not seem to be adequately explained by delays in commencement of passive rest or by other recorded methodological factors.

It is also intuitive to expect individual characteristics of personnel to contribute to inter-dataset variability in T_c_ cooling rates; however, their impacts during passive rest differ markedly from their impacts during work bouts. Participant age was restricted within this review due to age-based impairments of heat dissipation. Yet, such impairments primarily manifest during physical activity, resulting in higher T_c_ that are not adequately countered by heat loss during rest periods [73]. Similarly, participants undertaking fixed workload exercise with diminished physical fitness [74] or inferior heat acclimatisation [75] require longer to return their T_c_ to a given value during passive rest post-work, as a result of their insufficient heat dissipation during exercise rather than during the rest period per se. Hydration status was not reported for datasets within this review due to a lack of congruence between blood-based and urinary markers during physical activity, with the former infrequently utilised but nonetheless recognised for their validity [76]. As for age, fitness, and heat acclimatisation, hydration status primarily moderates heat exchange *during physical activity* and has less influence during rest periods [65]. Additionally, physical characteristics, through their influence on the thermal gradient between the skin and environment, influence T_c_ cooling rates in cold environments, particularly immersion in cold water [77]. Yet, across the 37 datasets where passive rest was undertaken with low clothing insulation (WBGT 17.0–39.0 °C), there was no association between the rate of T_c_ change and individual characteristics. While the 13 datasets where passive rest was undertaken with high clothing insulation (WBGT 16.5–36.1 °C) demonstrated associations between the rate of T_c_ change and body mass, BMI and BSA, the small sample size limits interpretation. Overall, the impact of participant characteristics on the rates of T_c_ change during passive rest reported within this review are not clear.

While a standard classification of post-work T_c_ cooling rates does not exist, McDermott et al. [78] utilised the range of 0.078 °C min^−1^ to 0.154 °C min^−1^ as acceptable T_c_ cooling rates in the treatment of exertional heat stroke. Their premise was the reduction of T_c_ from 42.2 °C to 38.9 °C as rapidly as possible. As occupational T_c_ are rarely synonymous with heat stroke [79], this classification is not applicable for determining the suitability of T_c_ cooling rates identified in this review. Brearley and Walker [20] adapted the work of McDermott et al. [78] to classify the T_c_ cooling rates of firefighters. Based upon the rapid elevations of T_c_ reported during firefighting activities [80], limited recovery time between work bouts (typically 15 min), and the objective of reducing T_c_ to below 38 °C, they determined acceptable T_c_ cooling rates for that cohort were −0.070 °C min^−1^ to −0.100 °C min^−1^. Observations from only one dataset of this review [53] met that criterion. It is possible that the T_c_ cooling rate of that dataset was inflated due to the period of up to 10 min between physical activity cessation and passive rest commencement, effectively allowing additional time for metabolic rate to decrease following exercise and prevent the rising or stable T_c_ often reported during the initial minutes of post-exercise rest periods [81]. However, the respective T_c_ cooling rates of −0.013 °C min^−1^ and −0.020 °C min^−1^ reported for datasets with a similar delay [34,50] were substantially lower. The firefighter cooling classifications were applied to the datasets of this review due to the lack of alternative occupational standards. Given that the T_c_ cooling rates of the remaining 49 included datasets were deemed unacceptable, and that firefighting combines severely restrictive PPE, extreme heat exposure, and periods of high workload that manifest as higher incidence rates of heat-related harm when compared to industrial work [82], T_c_ cooling rate standards specific to industrial and other occupational settings (for example, military) are required. To provide perspective, reducing T_c_ by just 0.5 °C during a 15-min rest period would require a mean T_c_ cooling rate of −0.034 °C min^−1^. That T_c_ cooling rate was exceeded in only 10 of the 50 datasets included in the current review, and all of those 10 datasets were from participants wearing athletic attire or similar, rather than more-insulative work attire [83,84]. Given the generally superior T_c_ cooling rates for participants wearing athletic attire during rest periods, modification of occupational attire during rest breaks should be considered to augment body heat dissipation post-work, but even this may not be sufficient in many situations.

In the absence of superior T_c_ cooling rates during passive rest than those reported in this review, additional or extended rest periods may be required to achieve desired cooling in occupational settings. Within many industries, protracted workforce rest periods may not be considered feasible nor commercially viable [85], potentially requiring implementation of active cooling modalities to compensate for insufficient passive cooling. However, with the exception of firefighting, there is limited evidence regarding active workforce cooling methods [16]. Based upon ready access within some industries [9], air-conditioned rest areas are a logical starting point [86] but will not be practicable in some occupational contexts. Irrespective of the cooling method, T_c_ measurement is a requisite to, firstly, identify individual responses to work bouts [72], and, secondly, verify rest periods are adequately reducing T_c_, particularly in higher WBGT categories [70,71]. Monitoring of T_c_ and other physiological responses would also permit evidence-based trials of work-to-rest allocations [87] and cooling methods. Despite the recent proliferation of wearable devices, accurate, non-invasive assessment of T_c_ in work settings remains problematic [88,89]. Furthermore, worker self-monitoring via perceptions of body temperature during rest periods may not provide a valid surrogate for T_c_ measurement [90], particularly where chronic heat exposure may “normalise” the perception of heat stress [91]. Reliance upon organisational heat-related illness reports to determine the need for worker cooling may also be risky due to under-reporting [92], despite the prevalence of heat stress and heat strain symptoms [92,93,94,95,96] that are likely to worsen with climate change.

Should climate projections of higher WBGT [14,97] be realised, exposed workers would endure the dual threat of higher T_c_ for a given volume of work [98], and the potential for decreased heat dissipation during rest periods. In this scenario, the effectiveness of passive rest periods to offset elevations in T_c_ experienced during work bouts will become more marginal, further reducing their commercial viability, particularly when undertaken in occupational attire. The development or refinement of active cooling controls to limit body heat storage during work and augment the T_c_ cooling rates reported by this review are warranted.

Several limitations of this review and the literature that informed it need to be acknowledged. First, none of the included studies were conducted in cooler environments (WBGT < 16.5 °C), where passive rest would typically have greater effects in lowering T_c_. However, in such environments, heat stress is less likely and so additional cooling strategies will not as often be necessary. Exceptions may include situations where workers are enclosed entirely in PPE during tasks, creating heat stress within the microclimate formed within the PPE—an example would be structural firefighters. In such instances, if the external environment is cooler, passive rest with PPE largely removed may be quite effective in cooling the body; however, no studies were identified that investigated this. Women were underrepresented in the included datasets—only 13 of the 50 datasets included female participants. This may limit the application of the review’s findings to female workers. Inadequate reporting of participant fitness levels, acclimatisation, and hydration statuses and lack of standardisation of participant work intensity and delays in commencing passive rest following physical work affect the types and strength of conclusions that can be drawn from the available data sets. Furthermore, passive rest was undertaken within controlled indoor conditions for the vast majority of datasets (45 of 50 datasets). The natural airflow and diffuse solar radiation of shaded outdoor settings may alter the T_c_ cooling rates of this review. The systems used within the included studies to monitor environmental conditions and T_c_ were in many studies poorly described, and so the validity of those systems was unclear. Future research in this area should address this deficit. Finally, there was a lack of datasets investigating the impacts on T_c_ of repeated bouts of physical work followed by passive rest [99]. This may limit the applicability of the review’s findings for occupational settings, where such repeated bouts of work followed by rest across many hours (and so progressive accumulation of heat load) are more common than in athletic contexts.

## 5. Conclusions

Since periods of passive rest are often recommended for workers to counter rises in T_c_ experienced during physical work, this systematic review aimed to identify the post-work T_c_ cooling rates conferred by passive rest, across a range of environmental conditions. From 44 eligible studies providing 50 datasets, the evidence indicated that during passive rest undertaken after physical work across a range of Wet Bulb Globe Temperatures, T_c_ reduced very slowly, or in some instances not at all, and in some participants it even increased. The common use of passive rest as a key cooling strategy for workers must therefore be questioned, as these findings indicate passive rest does not reverse elevated T_c_ of heat-exposed workers wearing work attire or part-attire in a timely manner. Where reductions in T_c_ are achieved through passive rest, the rest time required to achieve these reductions (typically an hour or more) is likely to be cost-prohibitive for most organisations. While somewhat faster cooling results were achieved in some studies when athletic attire was worn during cooling, such a finding was not consistent across the available datasets, and switching to athletic attire for rest periods at work may not be feasible in many work contexts. The fact that workers typically undertake repeated bouts of physical work followed by rest breaks in a work shift is likely to mean inadequate cooling during rest breaks leads to a progressive accumulation of heat in the body. Climate projections of higher WBGT are anticipated to make worker cooling via passive rest even less attainable in future years. Additional strategies to reduce heat stress and heat strain and increase T_c_ cooling rates are therefore needed both now and in the face of climate change to ensure workers do not suffer hyperthermia, heat illness, or heat stroke when physically active in hot and humid conditions.

## Figures and Tables

**Table 1:** Classification of WBGT (°C).

WBGT Category	WBGT Index (°C)
1	<27.7
2	27.8–29.4
3	29.5–31.1
4	31.2–32.2
5	>32.2

**Table 2:** Characteristics and key data of included studies.

WBGT Heat Category	WBGT(°C)	*n*	Mean Age (Years)	Mean Height (m)	Mean Body Mass (kg)	Mean Body Fat (%)	Mean BMI (kg/m^2^)	Mean BSA (m^2^)	Clothing	Clothing Insulation Factor (clo)		Time to Passive Rest (min)	Rest Duration (min)	Mean Pre T_c_	Mean Rate of ΔT_c_ (°C min^−1^)	Mean Time to Lower T_c_ 1 °C (mins)	Reference
										<0.4	≥0.4						
1	16.5	8M	24.8	1.81	73.0	14.1	22.4	1.93	Bunker Pants		●	-	40.0	38.8	−0.015	66.7	[61]
1	17.0	9M	24.1	1.80	79.5	-	24.5	1.99	Athletic attire	●		0	25.0	38.6	0.028	-	[40]
1	17.6	12M	21.8	1.84	80.1	-	23.7	2.03	Athletic attire	●		<5	30.0	38.8	−0.042	23.8	[48]
1	19.5	12M	21.3	1.83	76.2	-	22.8	1.98	Athletic attire	●		<5	30.0	38.5	−0.031	32.3	[28]
1	~21.0	5M	29.0	1.73	67.3	-	22.4	1.81	Underwear	●		-	75.0	39.0	−0.027	37.0	[41]
1	22.6	10M	25.6	-	80.3	-	-	-	Athletic attire	●		-	30.0	38.7	−0.028	35.7	[45]
1	22.8	7M 5F	24.0	1.71	71.4	19	24.4	1.83	Athletic attire	●		2	60.0	38.5	−0.016	62.5	[38]
1	23.0	6M	22.1	1.80	80.6	-	24.9	2.00	Encapsulated suit		●	0	30.0	38.7	−0.002	500.0	[64]
1	23.3	9M 9F	24.6	1.71	67.6	19	23.1	1.79	Athletic attire	●		2	60.0	38.5	−0.019	52.6	[37]
1	23.8	22M	24.0	1.76	70.7	-	22.8	1.86	Underwear	●		5	30.0	39.5	−0.060	16.7	[59]
1	24.7	15M 10F	26.5	1.74	72.7	16.2	23.9	1.87	Athletic attire	●		<5	60.0	38.8	−0.018	55.6	[31]
1	24.9	10M 7F	-	1.75	70.4	-	23.0	1.85	Athletic attire	●		3	15.0	39.0	−0.040	25.0	[29]
1	25.0	5	25.0	1.77	76.8	-	24.5	1.94	Athletic attire	●		0	62.0	39.4	−0.029	34.5	[54]
1	25.8	9M	21.0	1.83	78.7	-	23.5	2.01	Athletic attire	●		<10	20.0	38.5	−0.020	50.0	[50]
1	26.0	10M	21.0	1.76	76.0	-	24.5	1.92	Athletic attire	●		0	30.0	38.6	−0.019	52.6	[52]
1	26.2	9M 7F	24.0	1.82	74.0	17.1	22.3	1.95	Athletic attire	●		-	30.0	38.8	−0.034	29.4	[36]
1	26.5	5M 4F	25.1	1.74	75.4	-	24.9	1.90	Gridiron uniform		●	0	30.0	38.7	−0.032	31.3	[46]
1	27.0	14M 3F	28.0	1.80	68.5	11.2	21.1	1.87	Athletic attire	●		2–4	27.0	39.3	−0.060	16.7	[19]
1	27.3	10M	19.9	1.80	78.9	-	24.5	1.98	Athletic attire	●		<10	20.0	38.9	−0.070	14.3	[53]
1	27.4	6M	23.0	1.75	83.0	-	27.1	1.99	Athletic attire	●		0	60.0	38.8	−0.018	55.6	[44]
1	27.5	12M	24.0	1.72	-	11.7	-	-	Athletic attire	●		0	53.0	39.5	−0.040	25.0	[43]
2	28.0	10M	21.4	1.79	71.6	15.0	22.3	1.90	Athletic attire	●		0	30.0	38.9	−0.033	30.3	[39]
2	28.0	6M	22.1	1.80	80.6	-	24.9	2.00	Encapsulated suit		●	0	30.0	38.8	0.010	NA	[64]
2	28.8	9M 7F	26.0	1.76	72.5	20.7	23.4	1.88	Athletic attire	●		5	15.0	39.3	−0.040	25.0	[47]
2	29.0	15M	40.7	1.81	86.9	17.5	26.5	2.08	Firefighting uniform (lower body)		●	5.0	50.0	39.2	0.010	NA	[55]
2	29.1	8M	30.0	1.80	79.6	13.4	24.6	1.99	Athletic attire	●		0	20.0	39.9	−0.010	100.0	[66]
2	29.1	8M	30.0	1.80	79.6	13.4	24.6	1.99	Athletic attire	●		0	40.0	39.6	−0.012	83.3	[66]
2	29.4	8M	27.0	1.78	75.6	13.9	23.9	1.93	Athletic attire	●		1	90.0	39.2	−0.018	55.6	[65]
2	29.4	8M	23.0	1.77	81.4	14.7	26.0	1.99	Athletic attire	●		1	90.0	38.7	−0.008	125.0	[65]
3	29.5	15M	40.7	1.81	86.9	17.5	26.5	2.08	Firefighting uniform		●	5	20.0	38.2	0.010	NA	[56]
3	29.6	17M	23.8	1.77	79.4	-	25.3	1.97	Military uniform (lower body)		●	0	20.0	38.6	0.013	NA	[68]
3	29.7	18M	22.6	1.78	78.0	-	24.6	1.96	Military uniform (lower body)		●	0	20.0	38.6	0.008	NA	[68]
3	30.1	12	24.0	1.79	75.0	-	23.4	1.93	Athletic attire	●		0	20.0	38.8	−0.019	52.6	[32]
3	30.5	10M	22.0	1.71	65.0	-	22.2	1.76	Athletic attire	●		10	30.0	38.5	−0.013	76.9	[34]
3	31.1	8M	22.0	1.72	67.0	-	22.6	1.79	Athletic attire	●		-	15.0	38.6	0.000	NA	[51]
4	31.2	9M	24.0	1.77	76.7	14.7	24.4	1.94	Athletic attire	●		-	20.0	39.5	−0.014	71.4	[26]
4	31.3	11M 2F	23.0	1.77	78.6	19.6	25.2	1.95	Athletic attire	●		~5	15.0	39.1	−0.050	20.0	[30]
4	31.4	8M	21.4	1.72	61.8	-	20.9	1.74	Industrial Uniform		●	0	20.0	38.5	−0.010	100.0	[58]
4	31.5	13M 13F	23.8	-	71.2	19.4	-	-	Athletic attire	●		1	15.0	38.6	−0.053	18.9	[57]
4	31.7	10M	22.0	1.83	78.9	9	23.6	2.01	Gridiron uniform		●	0	30.0	39.8	−0.008	125.0	[49]
4	31.7	8M	25.0	1.81	86.7	16.5	26.5	2.07	Underwear	●			53.1	39.6	−0.030	33.3	[67]
4	31.7	6F	22.0	1.64	61.3	22.8	22.9	1.66	Underwear	●		1.7	29.0	39.5	−0.040	25.0	[67]
4	31.9	8M	25.0	1.81	86.7	16.5	26.5	2.07	Underwear	●		0	53.1	39.7	−0.030	33.3	[63]
4	31.9	6F	22.0	1.64	61.3	22.8	22.9	1.66	Underwear	●		1.7	29.0	39.5	−0.040	25.0	[63]
5	32.4	7M5F	26.0	1.71	76.0	18.5	26.1	1.88	Athletic attire	●		0	54.5	39.8	−0.028	35.7	[27]
5	32.5	12M	22.0	1.70	61.0	-	21.1	1.71	Industrial Uniform		●	0	30.0	38.6	−0.017	58.8	[33]
5	33.4	10M	22.0	1.71	62.0	-	21.2	1.73	Athletic attire	●		6	30.0	38.5	−0.018	55.6	[69]
5	34.1	10M	24.1	1.79	74.8	9.0	23.3	1.93	Military uniform		●	3	50.0	38.8	0.000	NA	[42]
5	36.1	10M	23.0	1.69	60.0	-	21.0	1.69	Industrial Uniform		●	6	30.0	38.5	−0.013	76.9	[60]
5	39.0	5M	25.0	1.77	82.4	-	26.2	2.00	Athletic attire	●		-	30.0	38.8	−0.014	71.4	[35]

BMI = body mass index, BSA = body surface area, kg = kilogram, m = metre, T_c_ = core temperature, WBGT = wet bulb globe temperature.

**Table 3:** Overview of datasets by WBGT category. Mean rest durations, pre-rest T_c_, and T_c_ cooling rates are weighted means, based on numbers of participants in the datasets contributing to each.

WBGT Category	WBGT Index (°C)	*n*	Mean Rest Duration (min)	Mean Pre-Rest T_c_ (°C)	Mean Rate of ΔT_c_ (°C min^−1^)	Estimated Mean Time to Lower T_c_ 1 °C (min)
1	<27.8	21	38.1	38.9	−0.027	37.0
2	27.8–29.4	8	42.9	39.2	−0.014	71.4
3	29.5–31.1	6	20.8	38.5	+0.002	Mean heating effect
4	31.2–32.2	9	25.8	39.2	−0.035	28.6
5	>32.2	6	38.4	38.9	−0.016	62.5

**Table 4:** Overview of *low* (clo < 0.4) clothing insulation datasets by WBGT category. Mean rest durations, pre-rest T_c_, and T_c_ cooling rates are weighted means.

WBGT Category	WBGT Index (°C)	*n*	Mean Rest Duration (min)	Mean Pre-Rest T_c_ (°C)	Mean Rate of ΔT_c_ (°C min^−1^)	Estimated Mean Time to Lower T_c_ 1 °C (min)
1	<27.8	18	38.6	38.9	−0.033	33.3
2	27.8–29.4	6	42.4	39.3	−0.023	43.5
3	29.5–31.1	3	22.0	38.6	−0.012	83.3
4	31.2–32.2	7	25.8	39.2	−0.041	24.4
5	>32.2	3	40.9	39.1	−0.022	45.5

**Table 5:** Overview of *high* (clo ≥ 0.4) clothing insulation datasets by WBGT category. Mean rest durations, pre-rest T_c_, and T_c_ cooling rates are weighted means.

WBGT Category	WBGT Index (°C)	*n*	Mean Rest Duration (min)	Mean Pre-Rest T_c_ (°C)	Mean Rate of ΔT_c_ (°C min^−1^)	Estimated Mean Time to Lower T_c_ 1 °C (min)
1	<27.8	3	33.5	38.7	−0.018	55.6
2	27.8–29.4	2	44.3	39.1	+0.010	Mean heating effect
3	29.5–31.1	3	20.0	38.5	+0.010	Mean heating effect
4	31.2–32.2	2	25.6	39.2	−0.009	111.1
5	>32.2	3	36.3	38.6	−0.010	100.0

## Data Availability

No new data were created or analysed in this study.
